# Ferritin's role in infectious diseases: Exploring pathogenic mechanisms and clinical implications

**DOI:** 10.1016/j.nmni.2025.101582

**Published:** 2025-03-20

**Authors:** Yingying Liao, Tao Zeng, Xiaoyan Guo, Xinhua Li

**Affiliations:** Department of Infectious Diseases, The Third Affiliated Hospital of Sun Yat-sen University, Guangzhou, 510630, China

**Keywords:** Ferritin, Hyperferritinemia, Biomarker, Infectious diseases, Inflammation

## Abstract

Ferritin, an iron storage protein, is crucial for maintaining iron metabolism balance throughout the body and serves as a key biomarker for evaluating the body's iron reserves. Reduced ferritin levels typically indicate iron deficiency, whereas elevated ferritin levels indicate an acute inflammatory response in infectious diseases. Recent research has established a significant link between elevated ferritin levels and disease severity and prognosis. The concept of hyperferritinemic syndrome has underscored ferritin's role as a pathogenic mediator. During infections, ferritin not only serves as a biomarker of inflammation but also exerts pro-inflammatory functions, which is a key factor in perpetuating the vicious pathogenic cycle. This review offers a comprehensive exploration of ferritin, covering its structural characteristics, regulatory mechanisms, and how diverse pathogens modulate ferritin. Understanding its pivotal role in infectious diseases is essential for identifying novel therapeutic prospects and enhancing disease management and prevention.

## 1 Introduction

Infectious diseases pose significant global health challenges, especially in low- and middle-income countries [1]. In 2019, approximately 13.7 million deaths worldwide were related to infections, highlighting their severe impact on global mortality [2]. Diseases like tuberculosis (TB) and emerging viral infections such as the coronavirus disease 2019 (COVID-19) have further strained healthcare systems [1]. In recent years, cost-effective strategies for the early identification of severe cases and the development of effective therapeutic approaches have gained increasing attention.

During an infection, both the host and the pathogen need to maintain access to iron for cell survival and proliferation [3]. Alterations in serum and cellular iron have been reported as important markers of pathology [3]. Ferritin, a readily accessible biomarker, has been extensively studied for its important role in iron metabolism and its potential as a critical indicator of infectious disease. This protein is vital in iron homeostasis by facilitating iron storage and delivery [4,5]. Its expression is regulated by various factors, such as oxidative stress and inflammatory stimuli [5]. Consequently, ferritin levels are often increased in different diseases, including infections and inflammatory disorders [4]. In certain conditions characterized by extremely high ferritin levels, this molecule may be hypothesized to have a pathogenic role contributing to the inflammatory burden. This has led to the introduction of the concept of hyperferritinemic syndrome. Firstly defined in 2013 by Rosário et al. [6], this syndrome included macrophage activation syndrome (MAS), adult-onset Still's disease (AOSD), catastrophic antiphospholipid syndrome (cAPS), and septic shock. The common feature of these diseases is represented by a marked hyperferritinemia [4,7]. Subsequently, emerging research indicates that ferritin is not merely a biomarker but actively contributes to pathogenesis by amplifying inflammatory responses [7]. Despite the growing knowledge of ferritin's roles beyond iron homeostasis, its precise functions in the pathogenesis of infectious diseases and its pro-inflammatory properties require further investigation. Thus, we will not primarily focus on the iron metabolism during infection because a comprehensive overview has recently been provided by Gomes et al. [3], our review comprehensively analyzes ferritin's pro-inflammatory properties, offering new insights into its involvement in infectious diseases.

## 2 Ferritin and iron homeostasis

### 2.1 Structure of ferritin

Ferritin is composed of two distinct subunits: ferritin light chain (FTL, 19 kDa) and ferritin heavy chain (FTH, 21 kDa) [8]. The genes encoding these subunits, *FTL* and *FTH1*, are located on chromosomes 19q and 11q, respectively [8]. The two subunits share approximately 55 % sequence homology and possess a similar three-dimensional structure, consisting of four parallel and antiparallel helices (A–D) and a fifth shorter E-helix that forms a 60° angle with the other helices [8]. The FTH plays a crucial role in iron oxidation, converting ferrous iron (Fe2+) to ferric iron (Fe3+), while the FTL helps in iron storage [9]. The ratios of the two subunits vary depending on the organ and cell type. For instance, the FTL predominates in the liver, while the FTH is more abundant in the heart [8]. In physiological states, ferritin can store nearly 2000 iron atoms. When fully saturated, it can store up to 4500–5000 iron atoms [8,9].

### 2.2 Regulation of ferritin

**Fig. 1:**
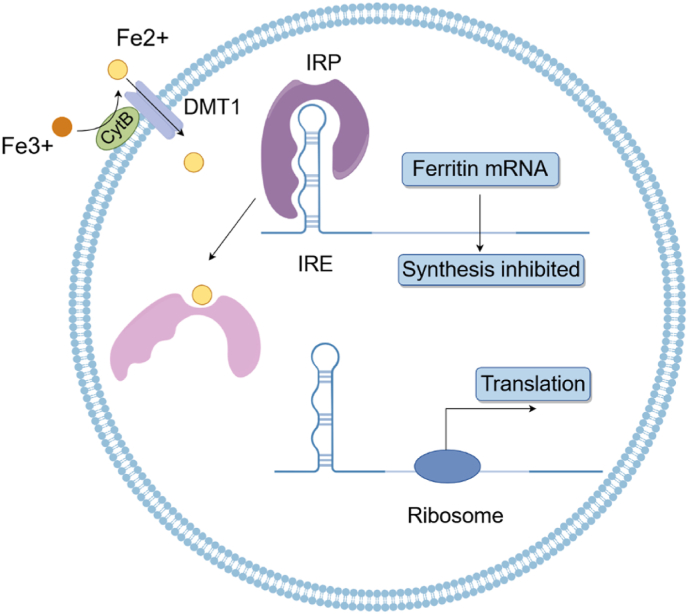
IRE/IRP regulation of ferritin. Extracellular Fe^3+^ is reduced to Fe^2+^ by CytB, which then enters cells via the DMT1. Under iron-deficient conditions, IRP binding to the IRE inhibits ferritin mRNA translation, thereby suppressing ferritin expression. Conversely, when intracellular iron content is increased, IRPs cannot bind to the DNA regulatory region of the IRE. This leads to the binding of the ribosome with the following expression of ferritin. Fe2+, ferrous iron; Fe3+, ferric iron; CytB, cytochrome B; IRE, iron-responsive element; IRP, iron regulatory protein; DMT1, divalent metal transporter 1.

The regulation of ferritin expression is very complex and involves translational level, transcriptional level, and post-transcriptional level [10]. Iron concentration is a key regulatory factor, with regulation primarily occurring at the post-transcriptional level [5,9]. Iron regulation mainly occurs through the interaction between iron regulatory proteins (IRP 1 and 2) and the iron-responsive element (IRE), a conserved 5′ structure on ferritin mRNA [11]. The IRE sensitively detects changes in intracellular iron levels and regulates ferritin expression accordingly [11]. Extracellular Fe^3+^ is reduced to Fe^2+^ by cytochrome B (CytB), after which it enters cells via the divalent metal transporter 1 (DMT1) [12]. Under iron-deficient conditions, IRP binding to the IRE inhibits ferritin mRNA translation, thereby suppressing ferritin expression. Conversely, elevated intracellular iron levels decrease IRP binding affinity to the IRE, promoting ferritin synthesis [11]. (Fig. 1).

Inflammatory stimuli also influence ferritin regulation [13]. Specifically, the cytokines such as tumor necrosis factor α (TNF-α), Interleukin-1 beta (IL-1β), and Interferon-gamma (IFN-γ) induce ferritin expression [13]. IL-1β facilitates ferritin translation by binding to a G and C-rich region in the 5′ UTR, common to the mRNAs of other acute phase proteins. Additionally, TNF-α and IFN-γ upregulate FTH mRNA expression at the transcriptional level through the nuclear factor-kappa B (NF-κB) signaling pathway [13].

**Fig. 2:**
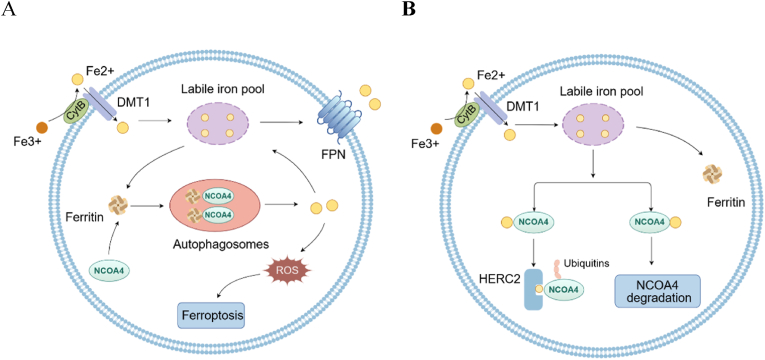
NCOA4-mediated regulation of ferritin. A At low iron concentrations, NCOA4 binds ferritin, leading to its degradation in autophagosomes and the release of iron. This leads to an increase in available intracellular iron (labile iron pool), as well as an increase in ROS. This mechanism of ferritin degradation (ferritinophagy) occurs in the iron-dependent pathway of cell death (ferroptosis). The released Fe2+ binds to FPN on the cell membrane and is then released into the bloodstream. B Under conditions of iron excess, ferritin collects free iron. Additionally, the regulatory protein HERC2 binds to NCOA4, promoting its ubiquitin-mediated degradation, along with basal autophagic degradation of NCOA4. NCOA4, nuclear receptor coactivator 4; ROS, reactive oxygen species; FPN, ferroportin; HERC2, HECT and RLD domain containing E3 ubiquitin protein ligase 2; CytB, Cytochrome B.

Ferritin degradation is another regulatory mechanism, involving a process called ferritinophagy [14]. Autophagy is a cellular process where cells degrade and recycle their components, and ferritinophagy is a specific type where ferritin is broken down to regulate iron levels [14,15]. This selective autophagic process is mediated by the binding of nuclear receptor coactivator 4 (NCOA4) to ferritin, essential for maintaining iron homeostasis [14]. The NCOA4-ferritin axis regulates intracellular iron content based on the cell's iron availability [14]. At low iron concentrations, NCOA4 binds ferritin, leading to its degradation in autophagosomes and the release of iron [5]. Under conditions of iron excess, the regulatory protein HECT and RLD domain containing E3 ubiquitin protein ligase 2 (HERC2) binds to NCOA4, promoting its ubiquitin-mediated degradation, along with basal autophagic degradation of NCOA4 [16]. Ferritinophagy can also lead to iron-regulated cell death, known as ferroptosis, which increases intracellular iron and reactive oxygen species (ROS), resulting in ferroptotic cell death [5,8]. The released Fe2+ binds to ferroportin (FPN) on the cell membrane and is then released into the bloodstream, where it can be used elsewhere in the body or by the cell for iron-dependent processes [17]. (Fig. 2).

## 3 Ferritin and the inflammatory response

Growing evidence suggests that ferritin is not merely an epiphenomenon of the acute-phase response but may actively contribute to the inflammatory process [4]. Following specific tissue injuries, pro-inflammatory cytokines stimulate the liver to produce ferritin, which is a multifunctional protein at the crossroads of immunity and inflammation [4]. This upregulation positions ferritin as both a consequence and mediator of inflammation.

**Fig. 3:**
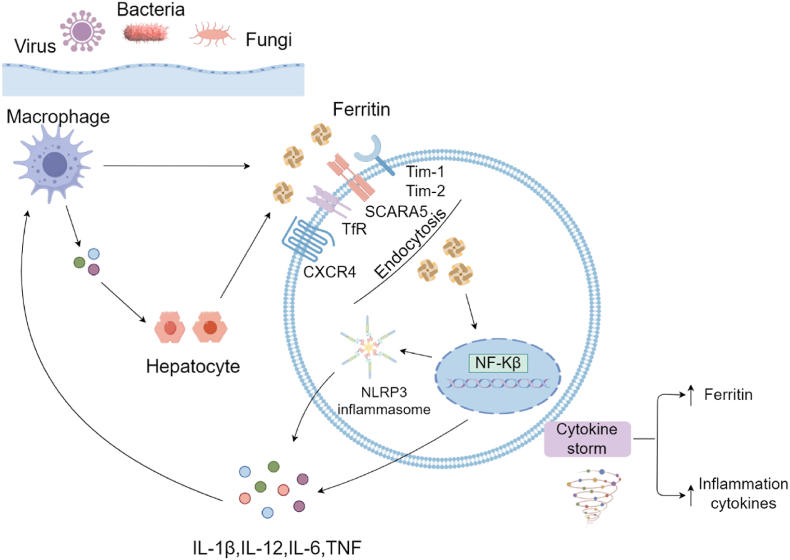
Several receptors have been identified in ferritin uptake, including TfR1, Tim1, Tim2 (in rodent models), CXCR4, and Scara5 (specifically for FTL). Upon binding to the cell surface, ferritin is endocytosed into the endosomes. while not all receptors are simultaneously required for endocytosis of ferritin, the schematic is demonstrating that all of the receptors in question have been found in the endosome. Inflammatory loop induced by high levels of ferritin. Pathogens result in the production of pro-inflammatory mediators, which in turn induce the production of ferritin. A vicious loop may be induced by ferritin and pro-inflammatory cytokines, including IL-1β, IL-6, and TNF, activating NF-κB and creating a pernicious cycle of inflammation that potentially escalates into a cytokine storm. FTL, ferritin light chain; TfR1, transferrin receptor 1; Tim-1, T-cell immunoglobulin and mucin domain-containing protein 1; Tim-2, T-cell immunoglobulin and mucin domain-containing protein 2; CXCR4, C-X-C motif chemokine receptor 4; Scara5, Scavenger receptor class A member 5; IL-1β, Interleukin-1 beta; IL-6, Interleukin-6; TNF, tumor necrosis factor; NF-κB, nuclear factor-kappa B.

However, the interplay between ferritin and inflammatory cytokines is bidirectional and intricate [18]. Ferritin can initiate and amplify inflammatory cascades, triggering the release of additional inflammatory cytokines [18]. A notable increase in serum ferritin levels indicates activation of the monocyte-macrophage system, a crucial component of the inflammatory cytokine storm [8]. In vitro studies provide compelling evidence for ferritin's causal role: upon FeH stimulation, cultured macrophages demonstrate increased expression of key pro-inflammatory cytokines, including IL-1β, Interleukin-6 (IL-6), Interleukin-12 (IL-12), and TNF [19]. The pro-inflammatory mechanisms of ferritin involve multiple receptors in ferritin uptake, such as Transferrin receptor 1 (TfR1) [9]. After endocytosis and binding to various receptors based on the FeH:FeL ratio, ferritin stimulates pro-inflammatory pathways independently of its iron content. This process ultimately activates NF-κB, resulting in increased inflammatory cytokine expression [20]. Moreover, ferritin functions as a damage-associated molecular pattern (DAMP), activating the NOD-like receptor protein 3 (NLRP3) inflammasome—a multi-protein complex critical to the innate immune response that promotes the maturation and release of pro-inflammatory cytokines [19]. (Fig. 3).

Consequently, ferritin and pro-inflammatory cytokines establish a self-perpetuating inflammatory cycle, where ferritin transitions from a consequence of initial inflammation to a driver of sustained immune activation. This process may escalate into a cytokine storm, increasing the risk of organ dysfunction [18].

## 4 Significance of ferritin in infectious diseases

Ferritin plays a critical role in inflammation, contributing to cytokine production, immune activation, and disease progression [7]. Increasing evidence highlights a strong association between elevated ferritin levels and various infectious diseases, particularly viral and bacterial infections [7]. Monitoring ferritin levels and understanding their significance can facilitate the timely implementation of effective therapeutic interventions for these diseases. The following sections of this review will explore the applications of ferritin in infectious diseases, examining its impact and utility from various perspectives.

### 4.1 Ferritin and viral infections

#### 4.1.1 Ferritin and viral hepatitis

Hepatitis B virus (HBV) is a hepatotropic DNA virus infecting millions globally, leading to chronic hepatitis B, liver cirrhosis, and hepatocellular carcinoma (HCC) [21]. In HBV-infected patients, normal iron homeostasis is significantly disrupted, resulting in hepatic iron accumulation that exacerbates liver injury and disease progression [22]. Specifically, the hepatitis B virus X protein (HBx), a critical Fe-S protein, induces ROS and upregulates FTH via IRP1 sensing changes in intra‐cellular iron level to regulate iron metabolism [23,24]. This molecular mechanism plays a fundamental role in the pathogenesis of HBV-associated liver diseases, substantially contributing to pathological progression in hepatic disorders [24]. Consequently, ferritin is closely associated with HBV infection-related lesions.

Serum ferritin may serve as a valuable prognostic marker for the progression and outcome of viral hepatitis. A retrospective study on the acute deterioration of HBV-chronic liver diseases (HBV-CLD) demonstrated that serum ferritin levels have high diagnostic accuracy for acute-on-chronic liver failure (ACLF) at admission, with an area under the receiver operating characteristic (AUROC) of 0.820 [25]. In this study, serum ferritin was found to be associated with liver and coagulation failure in patients with ACLF, and in patients without ACLF, it predicted the risk of 28-day progression to ACLF (AUROC: 0.808) [25]. However, its retrospective design and lack of external validation limit its clinical applicability.

Overall, ferritin functions as both a biomarker of disease progression and a contributor to liver pathology, highlighting its potential as a prognostic and therapeutic target.

#### 4.1.2 Ferritin and COVID-19

COVID-19, caused by the severe acute respiratory syndrome coronavirus 2 (SARS-CoV-2), primarily targets the lungs, presenting a range of clinical outcomes from asymptomatic conditions to severe cases characterized by pulmonary dysfunction and arterial hypoxemia, leading to acute respiratory distress syndrome (ARDS) [26]. Two hallmarks of severe COVID-19 are hyperinflammation and hyperferritinemia [7]. During the cytokine storm associated with COVID-19, numerous inflammatory cytokines, including IL-1β, IL-6, and IFN-γ are rapidly produced, stimulating ferritin secretion [18].

Serum ferritin serves as a significant predictive marker for COVID-19 patients, reflecting the severity, prognosis, and mortality. A retrospective single-center study of 141 patients revealed that elevated ferritin levels correlated strongly with disease severity, with significantly higher concentrations in severe cases compared to non-severe ones [27]. In addition, a meta-analysis identified serum ferritin as a predictor for progression to severe illness, indicating its potential role in risk stratification models for COVID-19 [28]. However, larger, multi-center studies with standardized protocols are essential to confirm ferritin's utility in evaluating COVID-19 severity. Furthermore, ferritin may act as a pathogenic mediator, enhancing inflammation and perpetuating the cycle of pathogenicity in COVID-19. It is a key mediator of immune dysregulation, exerting both immunosuppressive and pro-inflammatory effects that contribute to the cytokine storm [29].

Thus, emerging evidence suggests that ferritin may serve as both a biomarker of disease severity and an indicator of disease activity, helping to identify COVID-19 patients at risk for poor outcomes and hyperinflammatory responses. Moreover, it holds potential not only as a prognostic marker but also as a therapeutic target in severe COVID-19 cases.

### 4.2 Ferritin and bacterial infections

#### 4.2.1 Ferritin and Mtb

TB is a formidable global public health problem, causing over one million deaths annually [30]. It is caused by *Mycobacterium tuberculosis* (Mtb) and is transmitted through the inhalation of aerosols containing the bacteria. Consequently, TB primarily affects the lungs, serving as both the entry point and the main site of disease manifestation [31].

Mtb is phagocytosed by macrophages in the lung, where it persists and primarily replicates within these cells. Previous studies have demonstrated that primary mouse macrophages infected in vitro with *Mycobacterium avium* exhibit an increase in the expression of FTH [31]. Recent research has found that FTH and FTL are significantly increased in macrophages harboring live Mtb compared with uninfected macrophages or those harboring dead Mtb [32]. These findings suggest that the regulation of FTH plays a crucial role in mycobacterial infections. In addition, Mtb exploited the autophagic degradation of ferritin mediated by NCOA4, in macrophages for enhanced iron bioavailability and bacterial growth [32]. Thus, modulating host ferritin metabolism is identified as a novel Mtb strategy for intracellular growth, offering a potential target for host-directed therapy against tuberculosis.

Elevated ferritin has emerged as an important biomarker in TB progression. A case-control study conducted in two centers in China revealed significantly higher ferritin levels in patients with severe lung damage compared to those with mild or moderate lung damage due to TB [33]. Additionally, a prospective study further identified ferritin levels as a predictor of TB treatment outcomes, noting that a significant decrease in ferritin levels was observed only at the end of the 6-month anti-TB treatment period [34]. However, methodological limitations, including regional sample selection and the exclusive focus on active pulmonary TB cases, warrant validation in larger and more diverse cohorts.

Given its crucial role in Mtb disease severity and TB pathogenesis, ferritin serves not only as a key biomarker of TB progression but also as a promising indicator of therapeutic efficacy.

#### 4.2.2 Ferritin and sepsis

Sepsis is defined as a life‐threatening organ dysfunction caused by dysregulated host systemic inflammatory and immune response to infection, triggered by bacterial and other pathogenic microbial invasions [35]. Imbalances in pro-inflammatory and anti-inflammatory cytokines play a significant role in sepsis pathophysiology [36].

Ferritin demonstrates predictive value for sepsis severity and outcomes. Analysis of a large public database revealed nonlinear relationships between serum ferritin levels and clinical outcomes, with each 1000 ng/ml increase corresponding to elevated mortality risks of 13 %, 15 %, 16 %, and 17 % at 28 days, 90 days, 180 days, and 1 year, respectively [37]. However, the study primarily included critically ill patients, limiting the generalizability of these findings to broader sepsis populations. Additionally, in pediatric patients, multicenter investigations identified hyperferritinemic sepsis as a high-risk hyperinflammatory condition associated with increased mortality [38]. Further research is required to validate these findings in adult sepsis cohorts.

Clinically, ferritin serves as both a diagnostic and monitoring biomarker. In sepsis-related immune dysregulation, ferritin levels >4500 ng/mL demonstrate 98.0 % specificity and 97.2 % negative predictive value for diagnosing macrophage activation-like syndrome (MALS), a hyperinflammatory condition characterized by fever, hepatosplenomegaly, hepatic dysfunction, coagulopathy, cytopenias, and hypertriglyceridemia [36,39]. Importantly, a randomized clinical trial demonstrated improved 7-day outcomes with anakinra (IL-1 receptor antagonist) treatment in patients with ferritin levels >4500 ng/mL [36].

Understanding ferritin's dual role as both a biomarker and a contributor to sepsis pathogenesis may pave the way for more precise risk stratification and immunomodulatory interventions in sepsis treatment.

### 4.3 Hyperferritinemia and hyperferritinemic syndrome

Research indicates that serum ferritin levels exceeding 400 ng/mL are considered hyperferritinemia, a condition marked by excessive iron storage and frequently linked to severe inflammatory responses [40]. High ferritin levels are associated with a group of symptoms known as hyperferritinemic syndrome, including MAS, AOSD, CAPS, and septic shock [6]. These elevated ferritin levels are not merely a consequence of inflammation but may actively contribute to the development of cytokine storms, playing a significant pathogenic role [6]. In the context of AOSD, research reveals the intricate relationship between hyperferritinemia and the formation of Neutrophil Extracellular Traps (NETs). Ferritin has been found to stimulate the release of NETs through a Macrophage Scavenger Receptor 1 (Msr1)-dependent pathway, significantly contributing to systemic and hepatic inflammation. Consequently, this study highlights the important role of the ferritin-Msr1-NETs pathway in the overwhelming inflammatory response, serving as a therapeutic target against the spectrum of hyperferritinemic syndrome [41].

Recent research has revealed that patients with systemic lupus erythematosus (SLE), MDA5-positive dermatomyositis (MDA5+DM), severe COVID-19, and multisystem inflammatory syndrome (MIS) exhibit clinical and laboratory features similar to those of hyperferritinemic syndrome, with a strong correlation between ferritin levels and disease progression [4]. Consequently, it has been suggested that the scope of hyperferritinemic syndrome could be expanded to encompass severe diseases associated with hyperferritinemia and cytokine storms, including inflammatory, autoimmune, and infectious diseases [7]. These conditions, which may share common pathogenic mechanisms, often present with similar clinical features and could benefit from similar therapeutic approaches [6].

Studies indicate that decreased ferritin levels correlate with improved clinical outcomes, highlighting its therapeutic significance [42,43]. For example, intravenous immunoglobulin (IVIG) therapy lowers ferritin levels in sepsis and MAS, correlating with clinical improvement [6,44]. Additionally, Desferrioxamine (DFO), a well-established intracellular iron chelator, exhibits significant anti-inflammatory effects beyond iron sequestration, including the inhibition of NFκB [43,45]. In murine Campylobacter jejuni infection models, prophylactic oral DFO preserved intestinal barrier integrity, reduced apoptosis, and mitigated both local and systemic inflammation despite lacking direct antimicrobial effects [42]. Beyond iron chelation, alternative therapies target hyperferritinemia and inflammatory mediators. In cases of multiple organ failure (MOF) with disseminated intravascular coagulation (DIC) and hepatobiliary dysfunction, plasma exchange effectively eliminates excess ferritin and free hemoglobin, thereby disrupting the inflammatory cycle [43]. Further research is warranted to explore additional disease-specific therapeutic strategies and to refine the clinical application of ferritin-targeted therapies beyond iron chelation.

## 5 Discussion

Since the conceptualization of hyperferritinemic syndrome, mounting evidence suggests ferritin functions beyond a mere acute-phase reactant, acting as a pathogenic mediator and relevant clinical biomarker.

Pathogenically, ferritin may directly contribute to immune dysregulation by promoting pro-inflammatory mediator production during infection, primarily through modulation of iron metabolism, immune activation, and inflammatory cascades [4,46]. In viral infections, such as COVID-19, hyperferritinemia is a hallmark of severe disease, potentiating cytokine storms and creating a self-perpetuating cycle of inflammation [47]. Excess intracellular iron, mediated by NCOA4-driven ferritin degradation, generates ROS and triggers ferroptosis, exacerbating tissue injury [14,48]. Moreover, ferroptosis has been increasingly recognized as a key driver of viral pathogenesis [49]. Although the role of ferritin in ferroptosis has been explored, the precise mechanisms by which viruses interact with ferritin remain incompletely understood. Notably, in bacterial infections, humans sequester iron from pathogen sites as an innate defense strategy, known as nutritional immunity [50]. However, studies suggest ferritin may also supply iron to certain bacteria, such as Mtb [13]. Therefore, ferritin's role at the host-pathogen interface remains unclear for most pathogens, requiring further research. Although its exact functions in viral and bacterial infections are not fully elucidated, ferritin plays a crucial role in immune responses and iron homeostasis.

In conclusion, our study highlights ferritin's dual role in inflammation and disease progression. It serves as both a biomarker of inflammatory activity and a pathogenic mediator. Serum ferritin monitoring in clinical practice is warranted, given the correlation between elevated levels and disease severity. However, the exact role of ferritin, its specific implications in the cause of inflammatory and infectious diseases, and the mechanisms through which it correlates with disease severity remain incompletely elucidated. Despite the potential of ferritin-targeted therapies, optimal intervention thresholds have yet to be defined. Future research should clarify ferritin's pathogenicity and clinical significance to improve treatment of infectious and inflammatory diseases.

## CRediT authorship contribution statement

**Yingying Liao:** Writing – original draft. **Tao Zeng:** Writing – original draft. **Xiaoyan Guo:** Conceptualization. **Xinhua Li:** Writing – review & editing, Supervision.

## Declaration of competing interest

The authors declare that they have no known competing financial interests or personal relationships that could have appeared to influence the work reported in this paper.
